# Umbilical Cord Patch Transplantation for Corneal Perforations and Descemetoceles

**DOI:** 10.1155/2017/2767053

**Published:** 2017-06-04

**Authors:** Hua-Tao Xie, Dan Zhao, Yang Liu, Ming-Chang Zhang

**Affiliations:** ^1^Department of Ophthalmology, Union Hospital, Tongji Medical College, Huazhong University of Science and Technology, Wuhan 430022, China; ^2^Schepens Eye Research Institute, Massachusetts Eye and Ear, Harvard Medical School, Boston, MA 02114, USA

## Abstract

**Purpose:**

To evaluate the clinical outcome of umbilical cord patch (UCP) transplantation for deep corneal ulcers with perforations and descemetoceles.

**Methods:**

In this retrospective, noncomparative, interventional case series, 11 eyes of 11 patients with corneal perforation or descemetocele were included. The thickness and microstructure of UCP were measured. All eyes were treated with UCP and amniotic membrane transplantation for corneal reconstruction. Corneal ulcer healing, corneal thickness, anterior chamber formation, and best-corrected visual acuity (BCVA) were recorded and analyzed.

**Results:**

The thickness of human UCP is 398.6 ± 102.8 *μ*m (*n* = 5) with compact aligned fibers. The average age was 56.2 ± 15.8 (ranging from 22 to 75) years. The mean follow-up period was 7.1 ± 1.7 (ranging from 5 to 10) months. Four patients had descemetocele and 7 had perforation. The anterior chambers in all the 7 perforated corneas were formed at postoperative day 1. All patients regained a normal corneal thickness and smooth corneal surface within the first postoperative month. The vision improved in 10 eyes and remained unchanged in 1 eye. No recurrence nor side effects occurred during the follow-up.

**Conclusions:**

UCP can serve as an alternative material in the treatment of corneal perforations and descemetoceles. This treatment option is also beneficial in those countries with limited cornea donors and eye bank services.

## 1. Introduction

Corneal blindness is one of the three leading causes of blindness worldwide [1], with 10 million people having bilateral corneal blindness [2]. Corneal perforations and descemetoceles may result from a variety of inflammatory or infectious causes. Corneal transplantation is a definitive treatment for corneal defects [3, 4]. However, shortages of cornea donor remain a challenge worldwide [3, 4], especially in Asian countries such as China [5], although glycerol-preserved corneas largely overcome the lack of eye banking networks in developing countries [6].

Amniotic membrane (AM) transplantation has been used for corneal reconstruction over the past 20 years [7–11]. AM functions as a permanent graft or a temporary patch by promoting corneal epithelialization while suppressing stromal inflammation, angiogenesis, and scarring [7, 12]. AM of multiple layers is often used for deep corneal ulcer or perforation [8, 13–17]. However, it is difficult to handle due to the thickness and sticky surface. Moreover, the dislocation of the membrane or corneal leakage may occur after surgery [8] because of its weak tectonic support.

The umbilical cord patch (UCP) is an AM-UC-derived graft which is commercially available as AmnioGuard^®^, Bio-Tissue Inc., USA. Similar to AM, cryopreserved UCP also exerts therapeutic actions [18]. It is easier to handle [19] and has already been successfully used for the glaucoma shunt tubes covering [20] and conjunctival surface reconstruction [19].

We speculated that UCP may serve as a substitute material for the reconstruction of corneal perforations and descemetoceles. Herein, we retrospectively reviewed our clinical experiences of successfully treating 11 eyes of 11 consecutive patients by UCP transplantation.

## 2. Materials and Methods

### 2.1. Patients

This study was approved by the Ethics Committee of Wuhan Union Hospital, Tongji Medical College, Huazhong University of Science and Technology according to the tenets of the Declaration of Helsinki. We retrospectively reviewed 11 eyes of 11 patients with corneal perforations or descemetoceles treated with UCP transplantation at the Wuhan Union Hospital between November 2015 and June 2016.

### 2.2. Graft Preparations

Human AM [7, 21] and UCP [22] were prepared using previously described method with minor modification. Briefly, after written informed consent, human placentas with umbilical cords were obtained immediately after cesarean deliveries. Those positive for human immunodeficiency virus types 1 and 2, hepatitis virus B and C, and syphilis had been excluded. After washing with saline, the AM was separated from the chorion by blunt dissection and then cut into 3 × 3 cm piece. After removing the umbilical vessels and loose jelly tissues, the UCP was flattened and cut into 2 × 2 cm piece. Both AM and UCP were then washed 3 times with saline containing 50 *μ*g/ml penicillin, 50 *μ*g/ml streptomycin, and 2.5 *μ*g/ml amphotericin B before they were preserved in sterilized pure glycerin (Wuhan Union Hospital) at −20°C for at most 3 months. Immediately before use, the membranes were thawed, washed off with glycerin, and then immersed in saline for 10 minutes. The thickness and microstructure of those membranes were also measured by the anterior segment optical coherence tomography (OCT, Carl Zeiss Cirrus, Dublin, CA) and confocal microscope (HRT 3, Heidelberg, Germany), respectively.

### 2.3. Surgical Procedures

**Figure 1:**
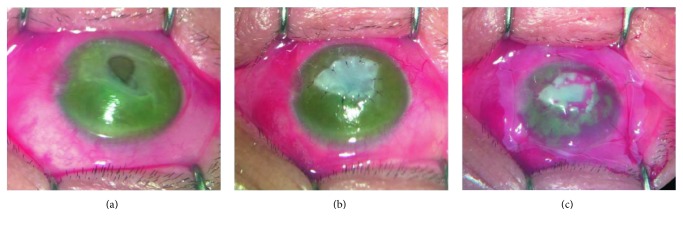
Surgical procedures. After the base and surrounding of the ulcer were cleaned of the necrotic tissue (a), the UCP with epithelium side facing up was trimmed to fit the shape and depth of the ulcer and interrupted sutures were placed to anchor the UCP to the cornea (b). Finally, a large piece of the AM with epithelium side up was applied over the entire cornea (c).

After topical and peribulbar anesthesia, the base and surrounding of the ulcers were cleaned of the necrotic tissue (Figure 1(a)). The UCP with epithelium side facing up was trimmed to fit the shape and depth of the ulcer, and interrupted sutures were placed to anchor the UCP to the cornea (Figure 1(b)). Finally, a large piece of the AM with epithelium side up was applied over the entire cornea as a temporary patch and anchored with a running 10–0 nylon and 4 interrupted sutures to the perilimbal episclera (Figure 1(c)). Permanent partial tarsorrhaphy was performed in patients with exposure-induced corneal ulcer.

### 2.4. Postoperative Management and Follow-Up

For bacterial keratitis, broad-spectrum antibiotic eye drops were administrated immediately, and topical steroids were used 4 times per day with weekly tapering. For herpes simplex keratitis (HSK), topical antibiotics and steroids along with oral acyclovir were applied (5 × 400 mg daily for 1 month and 2 × 400 mg for further 5 months). For exposure keratitis, topical antibiotics and steroids were administrated 3 times per day for 2 weeks. For fungal keratitis, all patients received 0.5% natamycin drops (Natacyn, Alcon Laboratories Inc., Belgium) 4 times daily for 4 weeks and oral itraconazole capsules (Sporanox, Janssen Ltd., Xi'an, China) 200 mg daily for 3 weeks. Topical 1% cyclosporin A (North China Pharmaceutical Co. Ltd.) was administered 3 times a day for 4 weeks.

All patients were followed up daily throughout the first week, biweekly for 1 month. Episcleral and corneal sutures were removed at around 1 week and 1 month after surgery, respectively. If patients had strong foreign body sensation after the episcleral suture removal, bandage contact lenses were applied. Clinical examination at each visit included best-corrected visual acuity (BCVA) and slit lamp (SL-2G, Topcon, Japan) examination. Fluorescent staining was used to monitor epithelialization, and anterior segment optical coherence tomography and confocal microscope were used to observe the postoperative recovery.

### 2.5. Statistics

All summary data was reported as mean ± SD calculated for each group and compared using Student's unpaired *t*-test using Microsoft Excel (Microsoft, Redmond, WA). The test results were reported as two-tailed *p* values, where *p* < 0.05 was considered statistically significant.

## 3. Results

### 3.1. Characteristics of the AM and UCP

**Figure 2:**
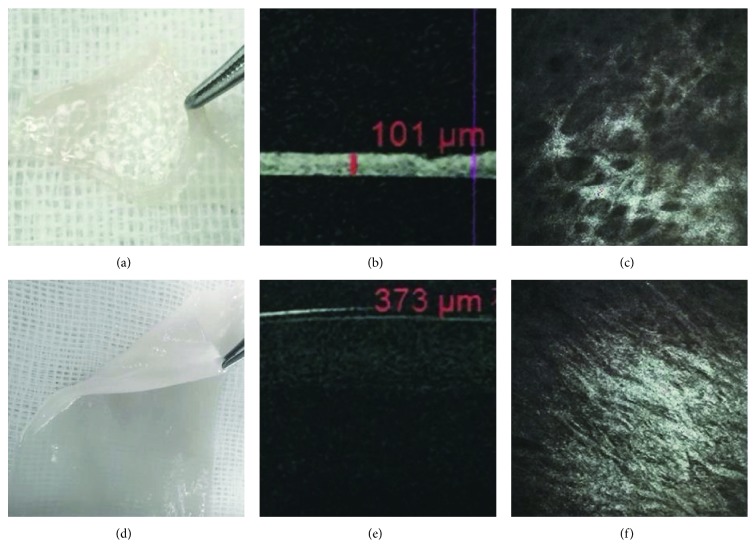
Characteristics of the AM and UCP. The thickness of human AM (a) is 97.6 ± 24.4 *μ*m (*n* = 5) (b) with loose fibers (c). The UCP (d) is much thicker (e) than the AM with a thickness of 398.6 ± 102.8 *μ*m (*n* = 5) (*p* < 0.001), and the stroma fiber is compact (f).

To detect the thickness and stromal microstructure of the AM and UCP, we used OCT and confocal microscopic scanning, respectively. The thickness of human AM (Figure 2(a)) was 97.6 ± 24.4 *μ*m (Figure 2(b)) with loose fibers (Figure 2(c)). The UCP (Figure 2(d)) is much thicker (Figure 2(e)) than the AM with a thickness of 398.6 ± 102.8 *μ*m (*p* < 0.001), and the stroma fibers are compact (Figure 2(f)). No cells were observed in their stroma (Figures 2(c) and 2(f)).

### 3.2. Patient Data and Therapeutic Outcomes

Relevant clinical data gathered from each patient are summarized in Table 1. There were 11 eyes in 11 patients (8 men and 3 women). The average age was 56.2 ± 15.8 (ranging from 22 to 75) years. The mean follow-up period was 7.1 ± 1.7 (ranging from 5 to 10) months. Patients with bacterial keratitis (*n* = 1), exposure keratitis (*n* = 2), herpes simplex keratitis (HSK) (*n* = 6), and fungal keratitis (*n* = 2) were enrolled in this study. The corneal ulcers were located centrally (*n* = 5) or paracentrally (*n* = 6), and the ulcer diameter was 4.6 ± 1.6 × 4.7 ± 1.2 mm (ranging from 2 × 3 to 7 × 6 mm). Four corneas showed descemetocele and 7 corneas perforated. All the patients complained of pain, tearing, photophobia, and foreign body sensation before surgery except 1 exposure keratitis patient concomitant with trigeminal nerve palsy after acoustic neuroma resection. UCP transplantation was successful in the reconstruction of the perforation or descemetocele in all 11 eyes. The anterior chamber in all 7 perforated patients formed at postoperative day 1. AM bandages were removed at around 1 week in all patients. Bandage contact lenses were applied in 8 eyes after AM removal to reduce the foreign body sensation caused by corneal sutures. All corneal ulcers healed with a normal corneal thickness and smooth corneal surface at the first month visit. All the patients were absent of irritation symptoms, and no recurrence was observed at the last follow-up visit.

The vision improved in 10 eyes and unchanged in 1 eye after surgery. The BCVAs of 6 eyes with paracentral ulcers showed improvement of at least 2 lines. Of the remaining 5 eyes with central ulcers, 4 eyes improved from hand motion to counting fingers and 1 eye stayed unchanged at counting fingers.

## 4. Representative Cases

### 4.1. Reconstruction of Perforated HSK

#### 4.1.1. Case Number 4

**Figure 3:**
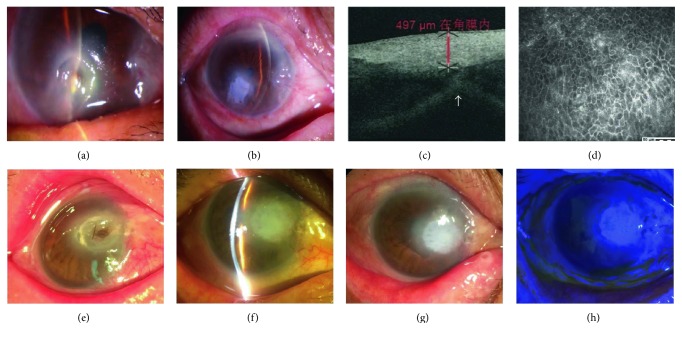
Reconstruction of perforated HSK. Case number 4. A 75-year-old female patient presented with a nonhealing corneal ulcer in her right eye caused by HSK. A deep corneal ulcer with a small perforation was noticed (a). One month after UCP transplantation, the anterior chamber formed (b) although a slight anterior synechiae of the iris was detected by OCT (c, arrow). The stromal thickness at the perforation site seemed normal (c). The UCP was completely epithelialized (d). Case number 6. A 70-year-old man had a 2 × 2 mm perforated corneal ulcer (e) secondary to HSK. One month after UCP graft, the cornea was successfully reconstructed with a well-formed anterior chamber (f). The cornea stayed stable (g) with a negative fluorescent staining (h) at the eighth-month visit.

A 75-year-old female patient presented with a nonhealing corneal ulcer in her right eye caused by HSK after a 28-day history of pain, tearing, photophobia, and foreign body sensation. There was a deep corneal ulcer in the lower part of the cornea, measuring 3 × 4 mm, with a small perforation (Figure 3(a)). One month after UCP transplantation, the anterior chamber formed well (Figure 3(b)) although a slight anterior synechiae of the iris was detected by OCT (Figure 3(c), arrow). The stromal thickness at the perforation site seemed normal (Figure 3(c)). The UCP was completely epithelialized (Figure 3(d)).

#### 4.1.2. Case Number 6

A 70-year-old man with a 2 × 2 mm perforated corneal ulcer (Figure 3(e)) secondary to HSK received UCP graft. One month after surgery, the cornea was successfully reconstructed with a well-formed anterior chamber (Figure 3(f)). The cornea stayed stable (Figure 3(g)) with a negative fluorescent staining (Figure 3(h)) at the eighth postoperative month.

### 4.2. Reconstruction of the Perforated Cornea with Fungal Keratitis

#### 4.2.1. Case Number 8

**Figure 4:**
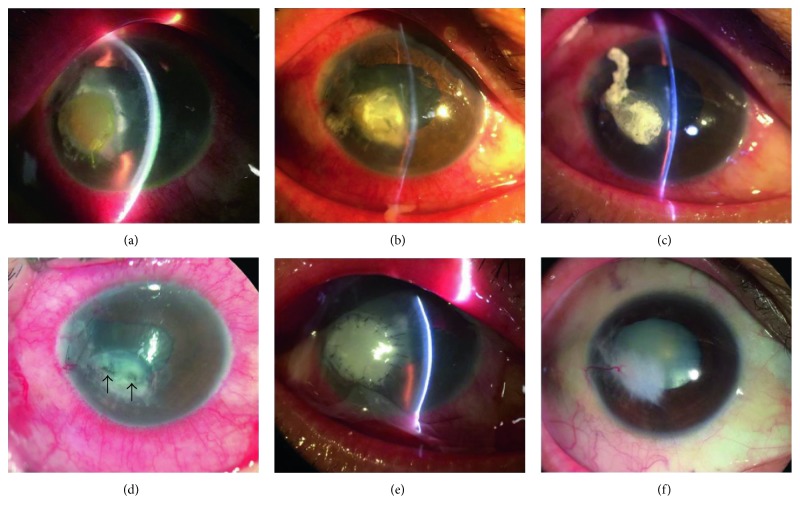
Reconstruction of the perforated cornea with fungal keratitis. Case number 8. A 46-year-old man suffered from severe ocular rupture in a trauma. One month after the suture of cornea, fungal corneal ulcer was observed (a). The ulcer area was gradually decreased at day 2 (b) and day 4 (c) after antifungal treatment. However, the anterior chamber became shallower (a, b, c). After removing the necrotic tissue, perforations were noted (d, arrows). The anterior chamber formed at postoperative day 1 (e). The cornea was successfully reconstructed, and a stable ocular surface without inflammation was achieved at the seventh month (f).

A 46-year-old man suffered from ocular rupture in a severe trauma accepted corneal suture. One month after surgery, corneal ulcer was observed around sutures and then the sutures were removed. Fungal hyphae and spores were found in the corneal smear immediately after his referral. Slit lamp examination revealed a paracentral cornea ulcer, with 6 × 6 mm in diameter (Figure 4(a)). The corneal ulcer area was gradually decreased at day 2 (Figure 4(b)) and day 4 (Figure 4(c)) after antifungal treatments. However, the anterior chamber became shallower (Figures 4(a), 4(b), and 4(c)). After removing the necrotic tissue during surgery, perforations were noted (Figure 4(d), arrows). The anterior chamber formed at postoperative day 1 (Figure 4(e)). The cornea was successfully reconstructed, and a stable ocular surface without inflammation was achieved at the seventh month (Figure 4(f)). The BCVA improved from hand motion to 20/200 even with traumatic cataract (Figure 4(f)).

## 5. Discussion

Our study demonstrated successful results in managing corneal perforations and descemetoceles in all 11 eyes with one procedure. The UCP is much thicker and compacter than the AM (Figure 2). These characteristics provide it with good toleration of sutures and full tectonic strength to achieve good sealing in all cases. None of the patients experienced dislocation, folding or dehiscence of the UCP, or corneal leakage. UCP also integrated into human corneal stroma with increased corneal thickness and full epithelialization based on OCT and confocal microscope results (Figures 3(c) and 3(d)). This excellent corneal compatibility is the same as previously reported on multilayer AM transplantation [23–25].

The ocular surface inflammation was markedly reduced with complete epithelialization in all patients at the first month visit. The therapeutic effect of UCP in managing deep ulcers and perforations may involve two basic actions that work synergistically in suppressing inflammation and promoting epithelialization [7, 12]. The cryopreserved umbilical cord was found to contain high quantity of biological signals, including high molecular weight hyaluronic acid (HA), heavy chain HA complex, and pentraxin 3 [18] which are recognized as the key relevant characteristic responsible for the AM's anti-inflammatory, antiscarring, and antiangiogenic effects [26]. The temporary AM that covered the entire cornea (Figure 1(c)) not only functioned as bandage contact lens to decrease the sense of irritation but also further augmented the effect of suppressing inflammation. These considerations are also important for patients with fungal infections (Figure 4(f)), in whom postoperative administration of steroids may result in fungal recurrence.

The BCVA analyses in our study demonstrated that all patients but one improved after UCP, of whom with paracentral ulcers showed improvement of at least 2 lines at the last follow-up. This indicated that UCP can serve as a definitive treatment in patients with paracentral lesions. The utilization of UCP is of great importance in those countries with a shortage of corneal tissues and eye bank services [3–5]. In cases of central corneal perforation, UPC can be transplanted as a first step to restore ocular integrity, control inflammation, and prevent infection. Then corneal transplant can be performed later to improve vision. This stepwise approach is preferred rather than performing tectonic corneal transplant at the time of perforation.

## 6. Conclusion

We have demonstrated the efficacy of UCP transplantation in repairing deep corneal ulcers with perforation or descemetoceles for the first time. Because of its easy availability and efficacy, the UCP is a significant alternative for corneal reconstruction.

## Figures and Tables

**Table 1:** Relevant clinical data of each patient.

Case	Age (yrs)	Sex	Eye	Primary diagnosis	Location	Diameter (mm)	Depth	BCVA		Follow-up (Mos)
								Before	After	
1	61	M	L	Bacterial keratitis	Paracentral	2 × 3	Descemetocele	20/400	20/40	10
2	22	F	R	Exposure keratitis	Central	4 × 6	Perforation	HM	CF	9
3	67	M	L	Exposure keratitis	Central	3 × 4	Perforation	HM	CF	9
4	75	F	R	HSK	Paracentral	3 × 4	Perforation	20/400	20/100	6
5	56	F	R	HSK	Central	5 × 5	Descemetocele	HM	CF	7
6	70	M	R	HSK	Paracentral	6 × 5	Perforation	HM	20/200	8
7	60	M	R	HSK	Central	7 × 6	Descemetocele	CF	CF	6
8	46	M	R	Fungal keratitis	Paracentral	6 × 6	Perforation	HM	20/200	7
9	53	M	L	Fungal keratitis	Paracentral	6 × 6	Perforation	HM	20/200	6
10	70	M	L	HSK	Central	3 × 3	Perforation	HM	CF	5
11	38	M	R	HSK	Paracentral	6 × 4	Descemetocele	20/400	20/50	5

BCVA: best-corrected visual acuity; CF: counting fingers; F: female; HM: hand motion; HSK: herpes simplex keratitis; L: left; Mos: months; M: male; R: right; yrs: years.
